# Effects of dezocine with ropivacaine on epidural analgesia during labor: a randomized controlled trial

**DOI:** 10.3389/fphar.2025.1586393

**Published:** 2025-04-28

**Authors:** Bin Su, Panlian Qian, W. P. Zhang, Yi Hou, Yiyi Shen

**Affiliations:** ^1^ Department of Anesthesiology, Tongxiang Maternity and Child Health Hospital, Jiaxing, China; ^2^ Department of Obstetrics and gynecology, Tongxiang Maternity and Child Health Hospital, Jiaxing, China; ^3^ Department of Anesthesiology, Women and Children’s Hospital, Jiaxing University, Jiaxing, China; ^4^ Department of Obstetrics, Women and Children’s Hospital, Jiaxing University, Jiaxing, China

**Keywords:** dezocine, epidural, labor analgesia, sufentanil, itching

## Abstract

**Background:**

Adding μ-opioid receptor agonists to local anesthetics are usually used for labor analgesia, while they are associated with pruritus. Kappa opioid agonists (dezocine) are widely used for pain management. Recently, they have emerged as a novel type of potent antipruritic agents. The purpose of this study was to investigate the effects of dezocine with ropivacaine on epidural analgesia during labor.

**Methods:**

A total of 120 parturients were randomly divided into two groups (60 cases each). The group D received 0.1% ropivacaine with dezocine 0.2 mg/mL for epidural analgesia while the control group received 0.1% ropivacaine with sufentanil 0.4 μg/mL for epidural analgesia. The systolic blood pressure (SBP), diastolic blood pressure (DBP) and heart rate (HR) were monitored. The onset time and duration of analgesia, pain intensity, Bromage scores, delivery outcome, neonatal Apgar scores, and adverse events of mothers and neonates were recorded. Pain intensity was assessed using visual analogue scale (VAS). Umbilical arterial blood was collected for analysis.

**Results:**

The incidence of itching was lower in the group D than the control group (0% vs. 10%, *P* = 0.036). The duration of analgesia was longer in the group D (76.1 ± 9.7 vs. 72.1 ± 10.5 min, *P* = 0.032), and numbers of boluses were fewer in the group D (median, 2 vs.3, *P* = 0.018). The onset time of analgesia and VAS values were similar between the two groups (all *P* > 0.05). There were no significant differences in terms of hypotension, bradycardia, motor block, respiratory depression, fetal acidosis, nausea and vomiting between the two groups.

**Conclusion:**

This study indicated that the epidural use of dezocine increased the analgesic effect, prolonged the duration of analgesia and decreased the incidence of itching during labor without increasing adverse events of mothers and neonates.

**Clinical Trial Registration:**

https://www.chictr.org.cn, identifier ChiCTR2000035341.

## Introduction

Local anesthetics in combination with μ-opioid receptor agonists are usually used for labor analgesia, while μ-opioid receptor agonists are associated with pruritus, nausea and vomiting (Cai et al., 2020; Cheng et al., 2019). Pain of the first stage is primarily visceral, which mainly resulted from uterine contractions (Labor and Maguire, 2008; Sandner-Kiesling and Eisenach, 2002). Kappa-opioid receptor agonists have been shown to be particularly effective analgesics in experimental models of visceral pain, these properties are expected to be of therapeutic interest in various visceral pain (Rivière, 2004). Kappa receptors are mainly distributed in the spinal cord (Harris et al., 2004; Rivière, 2004), and they are highly concentrated in the superficial layers of the lumbo-sacral spinal cord. While their density decreases in the upper levels of the spinal cord. The binding sites for kappa-opioid receptors are associated with visceral pain nociceptive inputs (Rivière, 2004). They are widely used for pain management (Koch et al., 2008; Gallego et al., 2007; Joppich et al., 2012; Tan and Conroy, 2018). Recently, kappa-opioid receptor agonists have emerged as a novel type of potent antipruritic agents. In 2021, the U.S. Food and Drug Administration approved difelikefalin (a kappa opioid agonist) for the treatment of moderate to severe pruritus associated with chronic kidney disease in adults undergoing hemodialysis treatment (Beck et al., 2023). Oxycodone, a κ and μ receptors agonist, was widely used for pain management. The literature demonstrated that epidural oxycodone could significantly prolong the duration of analgesia during labor. However, it might cause a higher incidence of pruritus (Zhong et al., 2020; Piirainen et al., 2019). Dezocine, a κ-receptor agonist and μ- receptor agonist-antagonist, is a synthetic, highly liposoluble, potent analgesic. It is now widely used for pain management (Zhou et al., 2017; Ye et al., 2022; Wang et al., 2018). It can produce analgesic effects by activating κ-receptor agonists as well as μ- receptor agonists. The adverse events of dezocine during the therapeutic process are primarily due to its partial activity at the μ opioid receptor. The adverse effects of κ-receptor agonists, including respiratory depression, pruritus, nausea and vomiting were less than those of pure μ-receptor agonists (Zhu et al., 2020; Zhou et al., 2015). At present, the literature on the use of dezocine for neuraxial analgesia is limited, especially in epidural labor analgesia. The aim of this study was to explore the effects of adding dezocine to ropivacaine on epidural labor analgesia.

## Materials and methods

This study was conducted in accordance with the Declaration of Helsinki and approved by the hospital ethics committee. Written informed consent was signed by all participants. The trial was registered at the Chinese Clinical Trials Registry (http://www.chictr.org.cn, registration number: ChiCTR2000035341). From July 2020 to May 2021, a total of 120 primiparous women were enrolled in this study. Inclusion criteria: age 20–35 years, height 152–175 cm, gestational age ≥37 weeks and weight 50–90 kg. Not-inclusion criteria: contraindications to epidural analgesia and cervical dilatation above 3 cm. Exclusion criteria: visual analogue scale (VAS) > 3 within the first 30 min after epidural administration and demanding cesarean section before administration of epidural analgesia. Parturients were randomly assigned to either the group D or the control group.

Randomization was carried out by opening an opaque, sealed envelope containing a sequential number. The allocation sequence was generated using random permuted block randomization. The investigators, anesthesiologists, obstetricians, and midwives were blinded to the study. Study drugs were prepared by a nurse who was blinded to this study.

On admission to the labor ward, non-invasive blood pressure (BP), heart rate (HR) and pulse oxygen saturation (SpO_2_) were routinely monitored at 10-minute intervals, and fetal heart rate was monitored continuously. Intravenous access was established. The epidural puncture was performed at the level of L_2–3_ interspace with an 18-G epidural needle using the method of decreasing of resistance to saline when the cervical dilation was about 2 cm, then an epidural catheter was inserted 4 cm cephalad into the epidural space. Five minutes after injection of a 3 mL test dose of 1.5% lidocaine, an initial volume of 10 mL of analgesic solutions was administered. The analgesic solutions contained 0.1% ropivacaine (AstraZeneca Pharmaceutical Co., Ltd., China) and dezocine (approval number:H20185140, Yangzijiang Pharmaceutical Co., Ltd, China) 0.2 mg/mL in the Group D, while the analgesic solutions contained 0.1% ropivacaine (AstraZeneca Pharmaceutical Co., Ltd., China) and sufentanil (Yichang Renfu Pharmaceutical Co., Ltd, China) 0.4 μg/mL in the control group. The analgesic solutions were prepared by a nurse who was blind to allocation and not involved in this study. If VAS values were >3 within the first 30 min after epidural administration, the women were excluded from this study. The analgesic solutions were continuously infused with an electronic infusion pump (Jiangsu Aipeng Medical Devices Co., Ltd. China). The parameters of the pump were set as follows: a background dose of 8 mL/h, a bolus dose of 6 mL and a lock time of 15 min. The women were given instructions on how to use an electronic infusion pump to give a bolus to relieve pain. A bolus of dose was administered when the VAS value was >3.

The onset and duration of analgesia, the duration of stages of labor, and delivery outcome were recorded. The blood pressure, heart rate and VAS were measured 5 min before analgesia (T0), 30 min after analgesia (T1), 3 cm of cervical dilatation (T2), 5 cm of cervical dilatation (T2), 7 cm of cervical dilatation (T2), and 10 cm of cervical dilatation (T5). Neonatal Apgar scores at 1 and 5 min were assessed. Umbilical arterial blood was sampled for analysis. Adverse events, including bradycardia, hypotension, itching, shivering, motor block, delivery modes, respiratory depression, nausea and vomiting, were also recorded. Hypotension was defined as a decrease in systolic blood pressure >20% of the baseline, and was treated with an intravenous injection of ephedrine 6 mg. Bradycardia was defined as a heart rate <60 beats per minute, and was treated with intravenous atropine 0.3 mg. Respiratory depression was described as an SpO_2_ < 91% and a respiratory rate <10 breaths per minute when receiving air. The pain severity was assessed with VAS (no pain: 0, mild pain: 1-3, moderate pain, 4-6, and severe pain:7-10) at 30 min intervals. The maximum level of sensory block was evaluated at 1-minute interval after drug injection using an alcohol cotton ball placed on skin. The onset time of epidural analgesia was defined as the time between drug administration and a T_10_ sensory block level being achieved, and was measured at 1-minute interval. The duration of epidural analgesia was defined as the time between the onset of T10 sensory block and VAS values above 3 following epidural analgesia. The motor block of lower limbs was assessed at 5-minute intervals using the modified Bromage score (Bromage 0 = fully able to flex knees and feet; Bromage 1 = just able to move knees; Bromage 2 = unable to move knees, able to move feet only; Bromage 3 = unable to move knees and feet). Motor block was considered as Bromage score >0. Itching was defined as an unpleasant and irritating sensation of the skin that provoked an urge to scratch or rub (Okutani et al., 2024). The severity of itching was assessed using a verbal rating scale: 0 = no itch, 1 = mild itch, 2 = moderate itch, and 3 = severe itch (Lockington and Fa’aea, 2007). A verbal rating scale of 1 or more was considered as itching.

### Statistical analysis

The primary outcome was the incidence of itching, the secondary outcomes were the onset and duration of epidural analgesia. According to our pilot study included 20 participants per group, the incidence of itching decreased from 15% to zero during epidural analgesia, we calculated that a sample size of 49 patients per group was sufficient to detect a difference in the incidence of itching between the two groups with a 2-sided alpha error of 0.05 and a power of 0.80. To account for dropouts, the sample size in each group was then increased to 60.

The results of our study were analyzed statistically using SPSS Statistics for Windows (ver. 22.0, SPSS Inc., US). The normality of the quantitative data was assessed using one-sample Kolmogorov-Smirnov test. Continuous variables were expressed as the mean ± standard deviation (SD) and analyzed using a *t*-test or analysis of variance. Non-normally distributed parameters were analyzed using the non-parametric Mann-Whitney U test. The categorical variables were expressed as numbers or percentages and analyzed using χ2 -test or Fisher’s exact test was used for categorical covariates. A *P* < 0.05 was regarded as significant difference.

## Results

**FIGURE 1:**
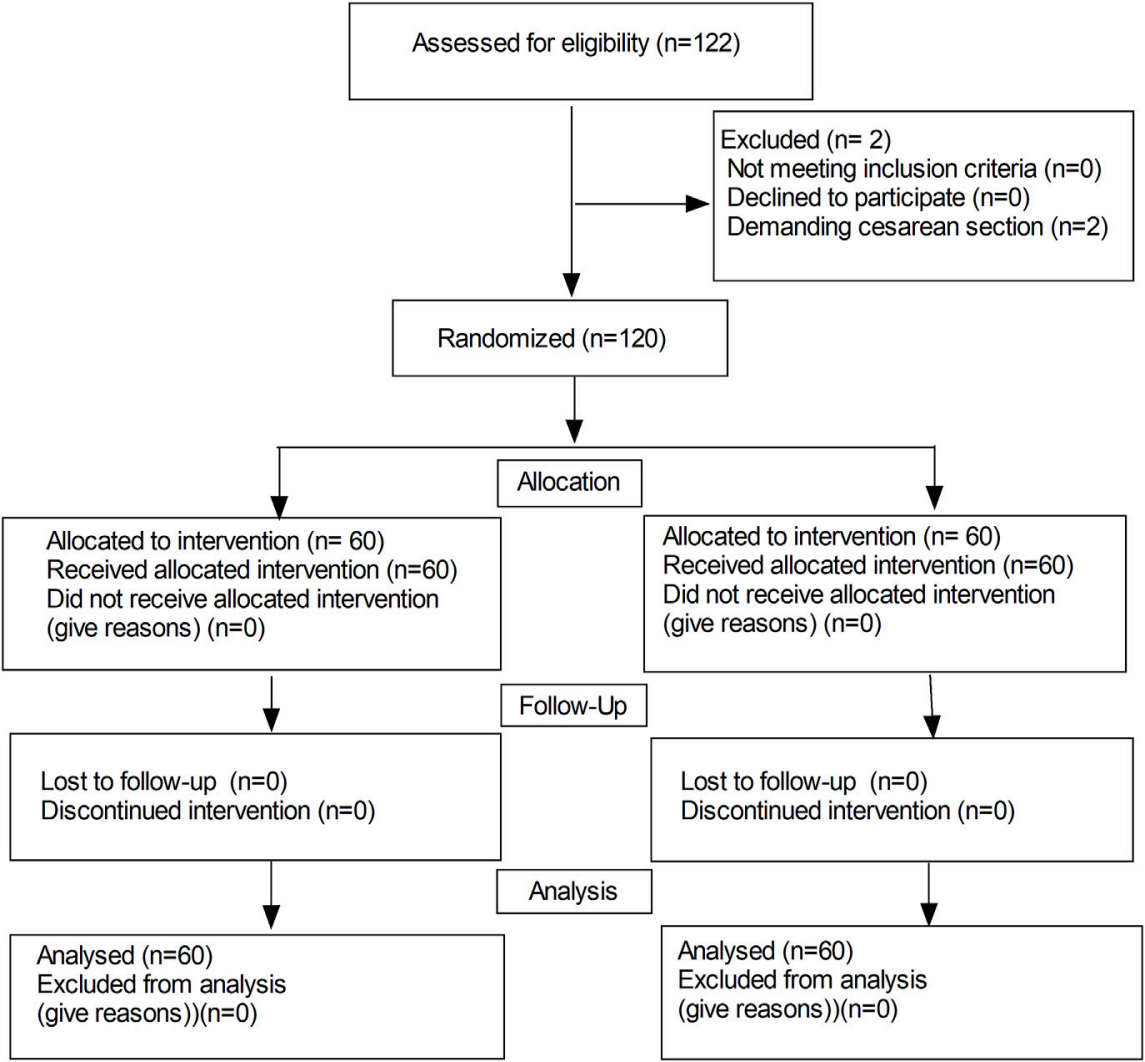
Flow diagram of study.

A total of 122 patients were initially enrolled in the study, 120 patients completed the study and 2 patients were excluded from the study due to demanding cesarean section before epidural analgesia (in Figure 1). No significant differences were observed in the maternal age, height, weight, gestational weeks, highest level of sensory block, onset time of analgesia, instrumental delivery, duration of the labor stages and neonatal Apgar scores between the two groups (in Table 1). While, there were significant differences in the duration of analgesia between the two groups (76.1 ± 9.7 vs. 72.1 ± 10.5 min, *P* = 0.032). Moreover, there was a significant difference in bolus numbers (median, 2 vs.3, *P* = 0.018) (Table 1).

**TABLE 1:** Data of women in both groups.

Variables	Group D (n = 60)	Control group (n = 60)	*P value*
Age (year)	27.6 ± 3.5	27.4 ± 3.7	0.724
Height (cm)	160.3 ± 3.9	159.9 ± 4.9	0.665
Weight (kg)	69.1 ± 7.1	70.9 ± 8.5	0.206
Gestational weeks (week)	39.3 ± 0.9	39.2 ± 0.8	0.436
Onset time of analgesia (min) Duration of analgesia (min) Highest level of sensory block (T6/T8)	10.3 ± 1.6 80.2 ± 7.9 24/36	10.1 ± 2.0 67.5 ± 7.7 25/35	0.393 0.032* 0.853
Duration of first stage of labor (min)	398.6 ± 81.6	403.9 ± 75.9	0.709
Duration of second stage of labor (min)	55.7 ± 14.1	57.6 ± 14.5	0.468
Duration of third stage of labor (min) Instrumental delivery	8.7 ± 2.7 2	8.9 ± 2.3 1	0.689 0.999
Numbers of boluses 1-min Apgar scores 5-min Apgar scores	2 [1-4] 9.0 ± 0.6 9.5 ± 0.6	3 [1-4] 9.0 ± 0.6 9.4 ± 0.6	0.018* 0.651 0.629

Data were presented as mean ± SD or numbers, *P < 0.05.

**FIGURE 2:**
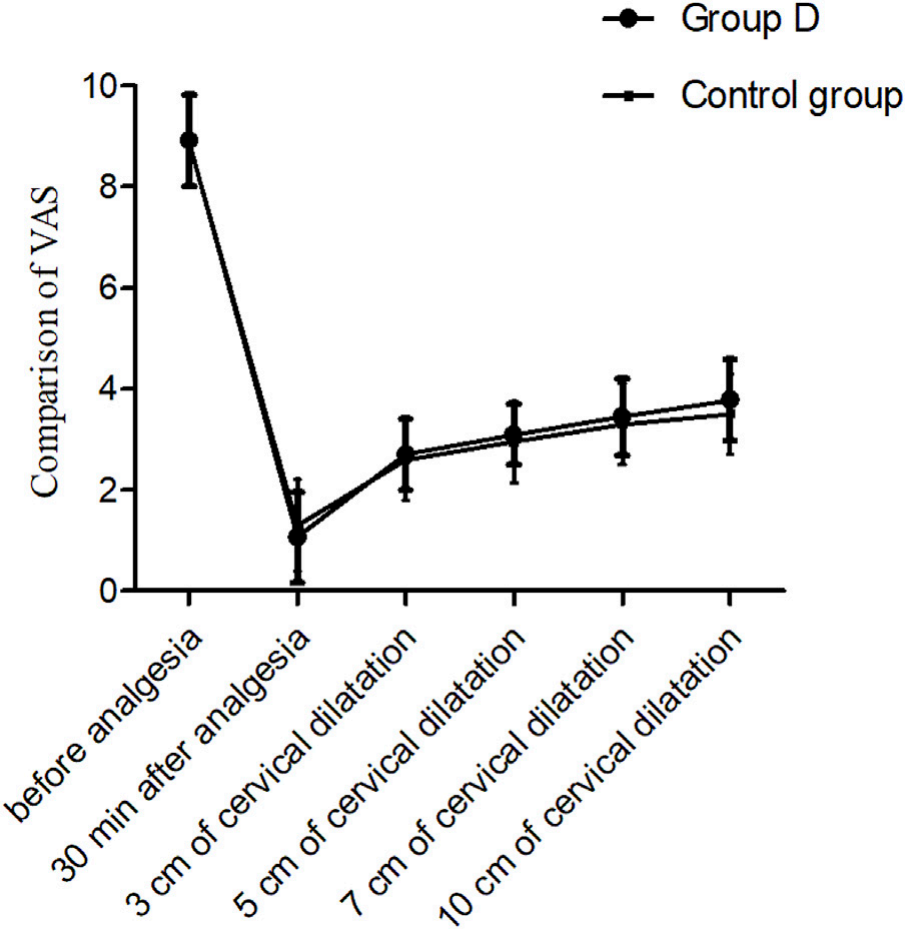
Comparison of VAS values at different time points, *P* > 0.05. T0: 5 min before analgesia, T1: 30 min after analgesia, T2: 3 cm of cervical dilatation, T3: 5 cm of cervical dilatation, T4: 7 cm of cervical dilatation, and T5: 10 cm of cervical dilatation.

The VAS values after epidural analgesia were significantly decreased than those before epidural analgesia in both groups (*P* < 0.01). While there were no significant differences in the VAS values after epidural analgesia between the two groups (all *P* < 0.05) (Figure 2).

**FIGURE 3:**
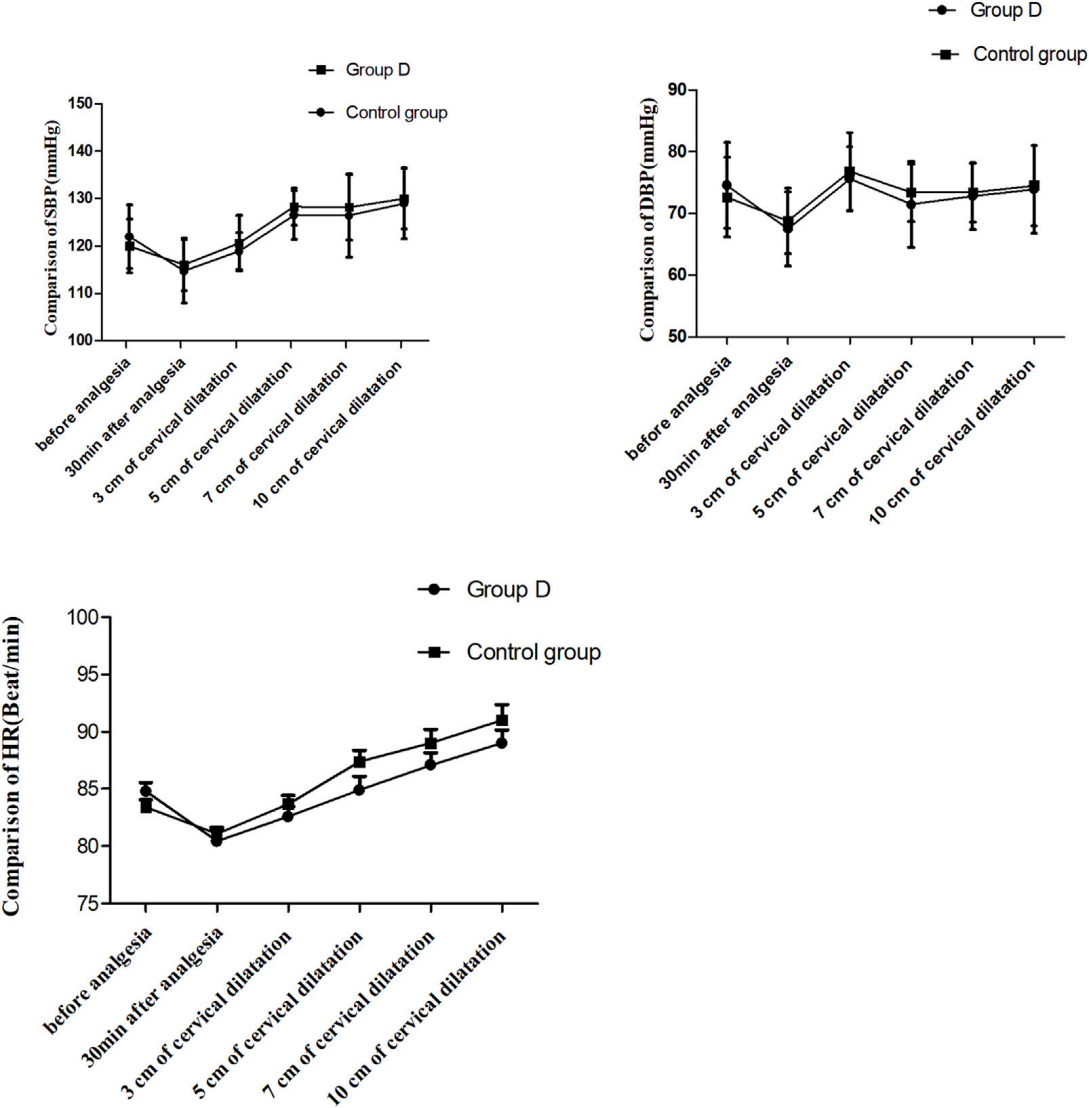
Comparison of blood pressure and heart rate at different time points, *P* > 0.05. SBP: systolic blood pressure, DBP: diastolic blood pressure; HR: heart rate. T0: 5 min before analgesia, T1: 30 min after analgesia, T2: 3 cm of cervical dilatation, T3: 5 cm of cervical dilatation, T4: 7 cm of cervical dilatation, and T5: 10 cm of cervical dilatation.

The systolic blood pressure (SBP), diastolic blood pressure (DBP) and heart rate (HR) are shown in Figure 3. The SBP, DBP and HR values after epidural analgesia were significantly decreased than those before epidural analgesia in both groups (*P* < 0.01). While there were no significant differences in SBP, DBP and HR at different time points after epidural analgesia between the two groups (*P* > 0.05).

The incidence of itching was lower in the group D (0% vs. 10%, *P* = 0.036), but the incidence of hypotension, bradycardia, motor block, respiratory depression, fetal acidosis, nausea and vomiting was similar between the two groups. No bradycardia, motor block, respiratory depression and fetal acidosis occurred in both groups (in Table 2).

**TABLE 2:** Adverse events of women and infants.

Index	Group D (n = 60)	Control group (n = 60)	*P -* value
Itching Vomiting and nausea	0 1	6 3	0.036* 0.611
Hypotension	2	3	0.999
Bradycardia	0	0	—
Motor block	0	0	—
Shivering Respiratory depression	2 0	3 0	0.999 —
Fetal acidosis	0	0	—

Data are shown as number, *P < 0.05.

## Discussion

In this study, we discovered that administering dezocine epidurally improved the analgesic effect, prolonged the duration of analgesia and decreased the incidence of itching during labor without increasing adverse events of the mothers and neonates.

The VAS values after epidural analgesia were significantly decreased in both groups (*P* > 0.05) compare with those before analgesia, and the VAS values were similar between the two groups. The present study shown that the analgesic effect of dezocine was the same as sufentanil when administered for epidural analgesia. There are two subtypes of μ receptors: μ1 receptor activation produces analgesic effects, while μ2 receptor activation is mainly related to adverse reactions, such as nausea, vomiting, respiratory depression, etc. (Pasternak and Pan, 2011). Dezocine mainly acts on κ receptors, which are mainly distributed in the spinal cord (Zhong et al., 2020). The κ receptor activation produces analgesic effects when dezocine acts on κ receptors of the spinal cord after epidural administration. In addition, dezocine possesses μ receptor agonist/antagonist effect. It produces the adverse effects related with activation of μ_2_ receptors when the activation of μ receptors (such as respiratory suppression, itching, nausea and vomiting, etc.). Moreover, it can antagonize the adverse effects caused by μ_2_ receptor activation when the activation of μ receptors. In present study, the duration of analgesia was longer in the group D (76.1 ± 9.7 vs. 72.1 ± 10.5 min, *P* = 0.032), and numbers of boluses were fewer. It indicated that dezocine could provide a longer duration of analgesia compare with sufentanil, as well as fewer bolus doses. Ning et al. (2024) found that the dezocine group showed lower visual analog scale scores, fewer mean boluses and lower incidence of adverse events when dezocine was used for patient-controlled epidural analgesia compared to the morphine group.

The SBP, DBP and HR values after epidural analgesia were significantly decreased than those before epidural analgesia in both groups (*P* < 0.05), but the incidence of hypotension (below 80% of baseline) was very low. No significant differences were observed in SBP, DBP and HR after epidural analgesia between the two groups. It showed that dezocine with low- concentration ropivacaine did not affect the stability of hemodynamics when administered for epidural labor analgesia. Chethanananda et al. (2017) also found that no significant variations occurred with respect to maternal hemodynamic parameters when fentanyl 2 μg/mL with 0.1% ropivacaine used for epidural labor analgesia.

In our study, there was no statistical differences in the duration of labor stages between the two groups, it indicated that dezocine did not prolong the duration of labor stages. Wang et al. (2015) found that there were no significant differences in the duration of labor stages between the use of ropivacaine alone and ropivacaine with sufentanil for epidural labor analgesia. Their results were similar to ours.

It is well known that opioid administration can cause itching. The incidence of opioid-induced itching differs with different opioids and routes of administration, and the various mechanisms can be broadly divided into peripheral and central (Okutani et al., 2024). The common side effects of μ-opioid receptor agonists are pruritus, respiratory depression, nausea and vomiting (Cai et al., 2020; Cheng et al., 2019). In this study, we found no itching when dezocine was administered epidurally for labor analgesia. The reason was that dezocine produced analgesic effect mainly by activating the κ receptor. The use of a multimodal analgesia combined with a mixed antagonist and κ agonists, especially μ-opioid antagonists, and κ-opioid agonists, seems to be the current best treatment modality for the management of opioid-induced pruritus and pain (Okutani et al., 2024). In this study, we found epidural dezocine did not increase the incidence of hypotension, motor block, respiratory depression, instrumental delivery, nausea and vomiting during labor. Dezocine can lead to respiratory depression by activating the κ receptor, but the incidence of respiratory depression is very low when used epidurally. Romagnoli and Keats (1980) proved that κ antagonist displayed limited sedative effect with a ceiling effecting for respiratory depression. Motor block is primarily associated with the concentration of epidural anesthetics. Sun et al. (2021) found that no serious adverse events directly associated with the analgesics were observed when nalbuphine with ropivacaine used for labor analgesia. Therefore, dezocine can be safely used for epidural labor analgesia.

This study found no respiratory depression and acidosis (umbilical artery pH < 7.2) in newborns. Besides, all the neonatal Apgar scores were more than 8. It indicated that dezocine could be used safely for epidural labor analgesia and did not increase the incidence of adverse effects of the newborns. Although dezocine can be excreted in breast milk, we found no respiratory depression in newborns.

### Limitation

Dezocine is not currently approved by the U.S. Food and Drug Administration (FDA) for epidural analgesia. The equivalent doses of two different opioids are unknown, these will affect the accuracy of the results. Further studies are required to evaluate the safety of dezocine during human pregnancy. The neonatal long-term outcomes were needed further investigation.

## Conclusion

This study indicated that the epidural use of dezocine increased the analgesic effect, prolonged the duration of analgesia and decreased the incidence of itching during labor without increasing adverse events of mothers and neonates.

## Data Availability

The original contributions presented in the study are included in the article/supplementary material, further inquiries can be directed to the corresponding authors.
